# Up-regulation of activating and inhibitory NKG2 receptors in allogeneic and autologous hematopoietic stem cell grafts

**DOI:** 10.1186/s13046-015-0213-y

**Published:** 2015-09-11

**Authors:** Alessandra Picardi, Andrea Mengarelli, Mirella Marino, Enzo Gallo, Maria Benevolo, Edoardo Pescarmona, Roberta Cocco, Rocco Fraioli, Elisa Tremante, Maria Concetta Petti, Paolo De Fabritiis, Patrizio Giacomini

**Affiliations:** Hematology, University of Roma Tor Vergata, Viale Oxford 81, 00133 Rome, Italy; Hematology, Regina Elena National Cancer Institute, Via Elio Chianesi 53, 00144 Rome, Italy; Pathology, Regina Elena National Cancer Institute, Via Elio Chianesi 53, 00144 Rome, Italy; Laboratory of Clinical Pathology, Regina Elena National Cancer Institute, Via Elio Chianesi 53, 00144 Rome, Italy; Laboratory of Immunology, Regina Elena National Cancer Institute, Via Elio Chianesi 53, 00144 Rome, Italy; Present address: Laboratory of Clinical Pathology, ASL Lanciano-Vasto-Chieti, Via Anello 66016, Guardiagrele, CH Italy

**Keywords:** Hematopoietic transplantation, hematopoietic malignancies, NKG2A, NKG2C, NKG2D, T lymphocytes, NK cells, HLA-E, MICA, ULBP

## Abstract

**Background:**

Hematopoietic Stem Cell Transplantation (HSCT) is known to induce the inhibitory immune receptor NKG2A on NK cells of donor origin. This occurs in allogeneic recipients, in both the haploidentical and HLA-matched settings.

**Methods:**

To gain further insight, not only NKG2A, but also the activating receptors NKG2C and NKG2D were assessed by flow cytometry. Immunophenotyping was carried out not only on CD56^+^ but also on CD8^+^ lymphocytes from leukemia and lymphoma patients, receiving both HLA-matched (n = 7) and autologous (n = 5) HSCT grafts. Moreover, cognate NKG2 ligands (HLA-E, MICA, ULBP-1, ULBP-2 and ULBP-3) were assessed by immunohistochemistry in diagnostic biopsies from three autotransplanted patients, and at relapse in one case.

**Results:**

All the NKG2 receptors were simultaneously up-regulated in all the allotransplanted patients on CD8^+^ and/or CD56^+^ cells between 30 and 90 days post-transplant, coinciding with, or following, allogeneic engraftment. Up-regulation was of lesser entity and restricted to CD8^+^ cells in the autotransplantation setting. The phenotypic expression ratio between activating and inhibitory NKG2 receptors was remarkably similar in all the patients, except two outliers (a long survivor and a short survivor) who surprisingly displayed a similar NKG2 activation immunophenotype. Tumor expression of 2 to 3 out of the 5 tested NKG2 ligands was observed in 3/3 diagnostic biopsies, and 3 ligands were up-regulated post-transplant in a patient.

**Conclusions:**

Altogether, these results are consistent with a dual (activation-inhibition) NK cell re-education mode, an innate-like T cell re-tuning, and a ligand:receptor interplay between the tumor and the immune system following HSCT including, most interestingly, the up-regulation of several activating NKG2 ligands. Turning the immune receptor balance toward activation on both T and NK cells of donor origin may complement *ex vivo* NK cell expansion/activation strategies in unmanipulated patients.

**Electronic supplementary material:**

The online version of this article (doi:10.1186/s13046-015-0213-y) contains supplementary material, which is available to authorized users.

## Background

Natural Killer (NK) cells bearing variable Killer Immunoglobulin-like Receptors (KIR) bind highly polymorphic class I Human Leukocyte Antigens (HLA-A,−B,−C), mount Graft versus Tumor (GvT) responses, and influence the outcome of patients with hematologic malignancies undergoing Hematopoietic Stem Cell Transplantation (HSCT). Unlike donor T cells, NK cells mediate limited Graft versus Host (GvH) reactions. Therefore, clinical transplantation protocols are being increasingly tailored on NK cells, in order to exploit a ligand:receptor mismatch of some kind (genetic and/or phenotypic) between any residual tumor cells in the recipient and the NK cell-repleted graft [1–3].

A full HLA haplotype mismatch occurs in haploidentical HSCT, with certain HLA-KIR combinations engendering optimal clinical effects. Similar mismatches may also arise in a fully HLA-matched allograft setting, since KIR segregate and are inherited independently from HLA. Even during hematopoietic self-replacement (autologous transplantation), approximately 60 % of the patients are expected to display the so-called ‘missing KIR’ phenotype, in that they express inhibitory KIRs that do not find a cognate HLA ligand on tumor cells. Although unlicensed and hyporesponsive, these NK cells may become cytokine-activated under appropriate conditions, positively affecting the outcome of hematopoietic malignancies and neuroblastoma [4, 5].

The genetic/phenotypic mismatch paradigm based on the variable KIRs is pervasive. However, both T and NK cells also express conserved (displaying little or no known polymorphism) receptors that sense changes in ligand expression associated with transformation and tumor cell stress. These may also find application in clinical transplantation. For instance, NKG2 receptors belong to a lectin-like protein family with inhibitory (NKG2A) as well as activating (NKG2C and NKG2D) members [6]. NKG2A and NKG2C bind the non-classical HLA class I molecule HLA-E. NKG2D binds the Major Histocompatibility Class I-like molecules A and B (MICA/B), and UL16 Binding Proteins (ULBPs). NKG2D ligands signal cellular stress in the un-manipulated host and, like HLA-E, they are overexpressed in tumor cells [7–11]. Conceivably, ligand overexpression and NKG2 receptor up-regulation may induce GvT through enhanced immune challenge, rather than overt donor-recipient mismatch.

To our knowledge, at least 7 studies [12–18] have examined NKG2 receptor expression in HSCT (summarized in Additional file 1: Table S1). There is general agreement that the inhibitory NKG2A receptor is up-regulated on immature, engrafting NK cells at an early education stage, whereas the triggering NKG2C and NKG2D receptors have been the subject of a few conflicting studies. NKG2D up-regulation was observed in 2/3 studies [14, 16, 17]. NKG2C up-regulation was seen in NK cells from cord blood [18] but not adult HSCT grafts [16]. Changes in NKG2 expression have never been correlated with engraftment kinetics, and the information available on autologous HSCT is limited to one study [12]. More in general, NKG2 receptors have never been assessed on CD8^+^ T cells (Additional file 1: Table S1). This is unfortunate, since cytotoxic T lymphocytes, like NK cells, integrate NKG2 receptor signaling in their lytic responses [19, 20]. Finallly, HLA-E, MICA/B, and ULBPs were never assessed on the tumors of transplanted patients. Thus, the phenotypic changes of NKG2 ligand:receptor pairs following HSCT are to our knowledge poorly defined, and their possible implications in GvT remain so far unappreciated.

To provide an overview of NKG2A, NKG2C and NKG2D up-regulation in different HSCT settings, on different immune effectors, in different hematologic malignancies, at different times post-transplant, and relative to engraftment, we have immunophenotyped both CD8 and CD56 lymphocytes from the peripheral blood of two series of representative recipients of HLA-matched allogeneic (*n* = 7) and autologous (*n* = 5) grafts. We have also assessed HLA-E, MICA, ULBP-1, ULBP-2 and ULBP-3 expression in 3 lymphoma specimens from autotransplanted patients. Results to be shown provide evidence for common, remarkably homogeneous post-HSCT phenotypic patterns, for the expression of NKG2 ligands on the tumor and cognate immune receptors on the lymphoid graft, and for phenotypic/clinical outliers among patients undergoing allotransplantation as well as autotransplantation.

## Methods

### Patients

The study was specifically approved by the competent Ethical Board (IRB 599/14). All patients and donors agreed to the research use of their diagnostic and biological specimens by signing an informed consent. Tables 1 and 2, as well as supporting information, summarize the features of the patients and treatments.

**Table 1:** Allotransplanted patients

Patient	Age	Diagnosis^a^	Donor^b^	Source of graft^c^	Conditioning regimen^d^	aGvHD^e^ stage	Onset of aGvHD (days)	Relapse (days)	Last follow up (days)	Status at last follow up	Cause of death
149	32	AML	U	PB	MAC	No	-	69	156	Dead	Relapse/Progression
150	26	DLBCL	R	PB	RIC	I	38	no	149	Dead	Infection
151	49	AML	R	PB	MAC	IV	12	124	293	Dead	Fungal Infection - Rejection/Poor graft Function^f^
152	33	B-ALL^g^	U	PB	MAC	III	88	no	137	Dead	Infection
153	37	SAA	R	PB + BM	MAC	II	40	no	307	Dead	Unknown
154	46	AML^h^	R	PB	MAC	No	-	299	365	Dead	Haemorrhage - CNS Toxicity
155	42	MM	R	PB	MAC	No	-	151	485	Dead	Relapse/Progression

^a^AML, acute myeloid leukemia; DLBCL, Diffuse Large B cell Lymphoma; B-ALL, B-cell Acute Lymphoid Leukemia; SAA, Severe Aplastic Anemia; MM, Multiple Myeloma

^b^Matched related (R) and matched unrelated (U) donor

^c^PB, peripheral blood; BM, bone marrow

^d^MAC, MyeloAblative Conditioning regimen; RIC, Reduced Intensity Conditioning

^e^aGvHD, acute Graft versus Host Disease

^f^remission induced by chemotherapy upon relapse (day 124)

^g^Patient previously diagnosed (year 1991) with CML t(9;22)

^h^Subclass III, according to the French American British classification of AML

**Table 2:** Autotransplanted patients

Patient	Age	Diagnosis^a^	Status at transplant^b^	Source of graft^c^	Conditioning regimen^d^	Relapse (days)	Last follow up (days)	Status at last follow up	Cause of death
187	60	AML	1st CR	PB	IBU	305	426	Dead	Relapse/Progression
188	19	DLBCL	1st PR	PB	BEAM	no	1159	Alive	
189	25	NHL	1st CR	PB	BEAM	no	1152	Alive	
190	50	HL	2nd CR	PB	BEAM	no	1149	Alive	
191	51	MM	1st PR	PB	MEL200	861	1239	Dead	Relapse/Progression

^a^AML, Acute Myeloid Leukemia; DLBCL, Diffuse Large B Cell Lymphoma; NHL, Non-Hodgkin’s Lymphoma; HL, Hodgkin’s Lymphoma; MM, multiple myeloma

^b^CR, complete remission; PR, partial remission

^c^PB: CD34^−^ peripheral blood autologous stem cells

^d^BEAM: Carmustine, Etoposide, Cytarabine, Melphalan; IBU: Idarubicine, Busulphan; MEL200: Melphalan

### Flow cytometry

Whole blood was collected in BD Biosciences vacutainer collection tubes K2E (EDTA) at room temperature. Plasma was removed by washing, and 100 μl aliquots of packed blood cells were pre-incubated with 1 μg of murine Ig to saturate Fc receptors, and then stained (two-color fluorescence) by simultaneous incubation for 30 min on ice with an FITC-conjugated mAb to either CD8 (clone DK25 from Dako) or CD56 (clone MEM-188 from Euroclone), and with PE-conjugated mAbs to NKG2A, NKG2C or NKG2D (clones 131411, 134591 and 149810, respectively, all from R&D systems). A mouse Ig isotype cocktail, and the CD4/CD8 simultest (both from BD Biosciences) were used for fluorescence calibration. At the end of the incubation, red blood cells were lysed by FACS lysing solution (BD Biosciences 92–0002) for 15 min at room temperature in the dark. Gated mononuclear cells were read and analyzed in a FACScan by CellQuest (BD Biosciences).

### Variable Nucleotide Tandem Repeat (VNTR) assay

Genomic DNAs were extracted from the Peripheral Blood Mononuclear Cells of both recipients and donors. Chimerism was assessed by Quantitative Real-Time PCR (QRT-PCR) amplification as described [21], and expressed as the percentage of donor DNA in sample DNA. For further details see Additional file 1: Materials and Methods.

### Immunohistochemistry

Immunohistochemistry was performed on all the available diagnostic biopsies from patients receiving autotransplants. Antigen retrieval (dewaxing, rehydration and heating) of Formalin Fixed Paraffin Embedded (FFPE) tissues, and staining with the HLA-E-specific mAb MEM-E/02 were performed as described [22]. Rabbit polyclonal antibodies used in this study were: ab93170 to MICA (AbCAM, Cambridge, UK), NBP1-80856 to ULBP-1, 27080002 to ULBP-2, and NBP2-31866 to ULBP-3 (Novus Bio, Littleton, CO, USA). As recommended by the manufacturers, antigen retrieval was carried out at pH 6.0 in all cases except that for ULBP-2 (pH 8.0). Since these antibodies are polyclonal, we validated their specificity on a panel of cell lines pre-tested by flow cytometry with mAbs to MICA and ULBPs [23, 24], and on FFPE tissues known to express these antigens, as indicated in the antibody leaflets. Sections of FFPE cell pellets and tissues were stained with a wide range of antibody dilutions, and for each antibody a working dilution was identified resulting in optimal staining of positive specimens with no detectable background on flow-cytometry-unreactive cell lines, as follows: antibodies to MICA and ULBP-1 (1:50); antibodies to ULBP-2 and ULBP-3 (1:500). Staining was revealed by a supersensitive streptavidin-biotin immunoperoxidase system (Biogenex, Menarini, Italy). Staining scores were: *0* (from undetectable to detectable staining in <10 % of tumor cells); *1* (>10 % but <50 %); *2* (>50 % but <80 %); *3* (>80 %).

## Results

### Up-regulation of NKG2A, NKG2C and NKG2D in CD8^+^ T lymphocytes and CD56^+^ NK cells from allotransplanted patients

**Fig. 1:**
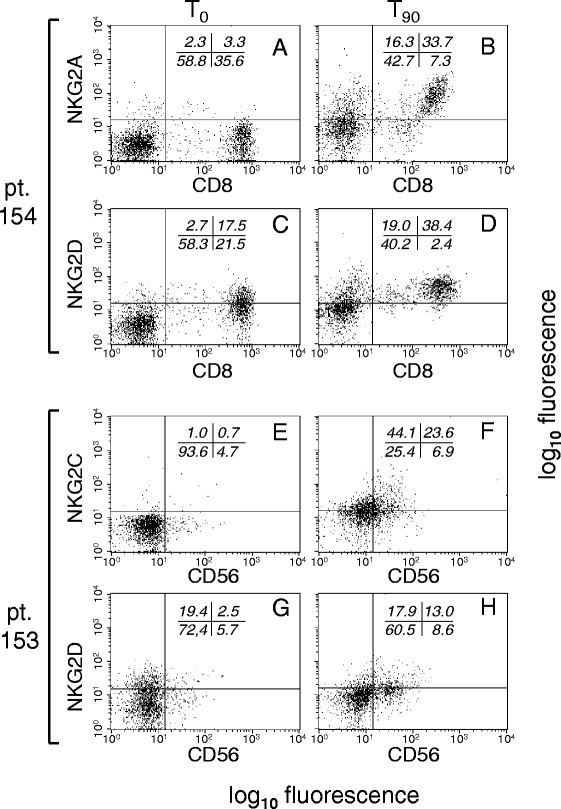
Flow cytometry evaluation of the expression of NKG2A and NKG2D in CD8^+^ and CD56^+^ WBCs from allotransplanted patients. WBCs obtained from patients 154 (**a-d**) and 153 (**e-h**) at the indicated times (relative to HSCT) were double-stained with mAbs to either CD8 or CD56 (abscissae), and to either NKG2A or NKG2D (ordinates)

Blood was obtained before and after transplant (at days 0, 30 and 90; T_0_, T_30_ and T_90_) from the 7 patients listed in Table 1, and from their respective HLA-matched donors prior to donation. In analogy with previous studies, and to permit comparisons with the available data, the levels of NKG2A (inhibitory), as well as NKG2C and NKG2D (activating) immune receptors were assessed on CD8^+^ and CD56^+^ cells by two-color flow cytometry. Representative results of CD8^+^ cells from patient 154 (Fig. 1a-d), and CD56^+^ cells from patient 153 (Fig. 1e-h) show that percentages and mean fluorescence intensities (mfi) values of NKG2A, NKG2C and NKG2D were simultaneously and drastically increased at T_90_. Increased NKG2 expression in CD8^−^ and CD56^−^ cells from both patients was also visible (Fig. 1b, d, f and h), demonstrating generalized up-regulation on both CD8^+^ and CD56^+^ cells from both patients.

**Fig. 2:**
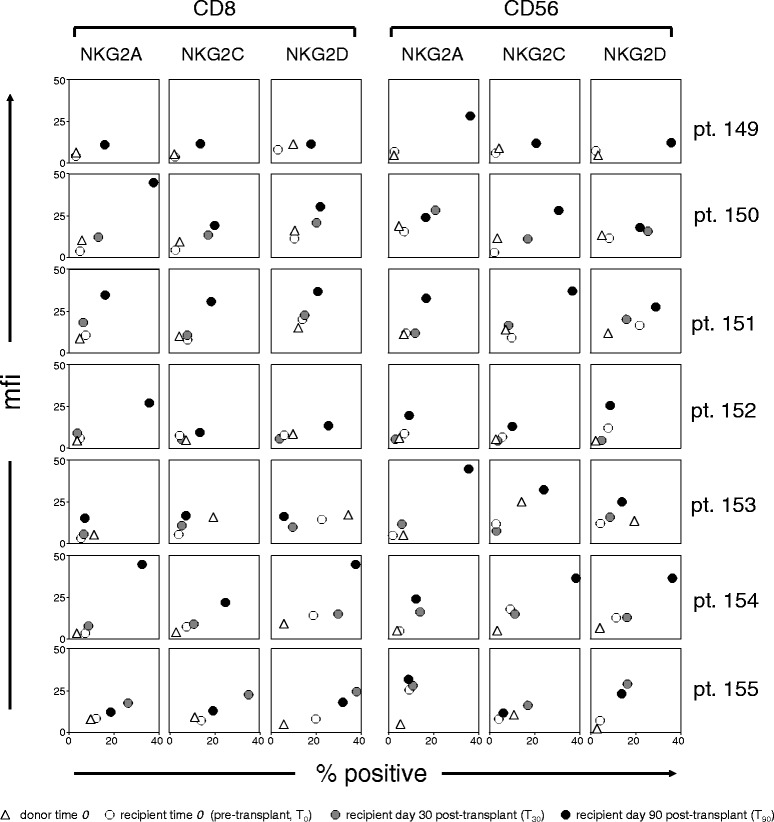
Quantitative evaluation of NKG2A, NKG2C and NKG2D levels at different times following allogeneic HSCT. Flow cytometry data obtained from 7 patients were graphically elaborated as described in the text. The results were expressed as a 2D plot (percent positives in abscissae and mfi in ordinates) separately displaying, for each patient, the time-course of the increases in NKG2A, NKG2C, and NKG2D levels, and the percentages of NKG2-positive CD8^+^ and CD56^+^ cells

This may be more clearly appreciated when the NKG2 expression data from all the patients and donors are graphically displayed (Fig. 2). As shown in this synopsis, the percentage of double-positives (CD8^+^/NKG2A^+^, CD8^+^/NKG2C^+^, CD8^+^/NKG2D^+^, CD56^+^/NKG2A^+^, CD56^+^/NKG2C^+^, and CD56^+^/NKG2D^+^) is plotted in abscissae, and the corresponding NKG2A, NKG2C, or NKG2D mfi value (calculated by taking into account the events in the upper and lower right quadrants) is plotted in ordinates. As a result, percent positives and mfi are factored into a single parameter, e.g. the distance (slant) from the plot origin: the greater is the distance, the greater is the up-regulation (Fig. 2). A slant value may also be calculated for each receptor and time point by multiplying the percent of positive cells and mfi values, as described in Additional file 1. Pearson’s correlation coefficients among series of NKG2A, NKG2B and NKG2C slant product values (Additional file 1: Table S2A), demonstrated highly significant correlations. Thus, the three NKG2 receptors are simultaneously up-regulated after transplant in most cases. From the synopsis in Fig. 2 and Additional file 1: Table S2A it may be concluded that: (a) NKG2A, NKG2C and NKG2D were expressed at very low levels in the pre-transplant CD8^+^ and CD56^+^ cells from both donors and recipients; (b) essentially all NKG2 receptors were up-regulated in both CD8^+^ and CD56^+^ cells from all the patients, with occasional selective enhancements in individual patients; (c) optimal up-regulation occurred at day 90 in all patients except pt. 155, displaying a more marked enhancement at day 30 than at day 90; (d) up-regulation occurred independently of, and with no obvious correlation with, either the clinical pathological features or the success in donor cell engraftment (see Table 1); and lastly (e) up-regulation also occurred in a patient transplanted for Severe Aplastic Anemia (pt. 153, see Table 1), e.g. a non-neoplastic hematologic condition, although with considerable potential for neoplastic evolution.

As reported previously [12–15], antibodies to KIR2DL1 and KIR2DL2, included as controls, detected either no change or a moderate decrease in expression (not shown). Finally, significant post-transplant increases in CD56 mfi values were also observed (T_0_ as compared to T_90_; *p* = 0.018 upon two-tailed paired Student *t* test; Additional file 1: Table S3), suggesting an overall immature phenotype of engrafting (see below) NK cells.

### Engraftment and NKG2A/C/D up-regulation

**Fig. 3:**
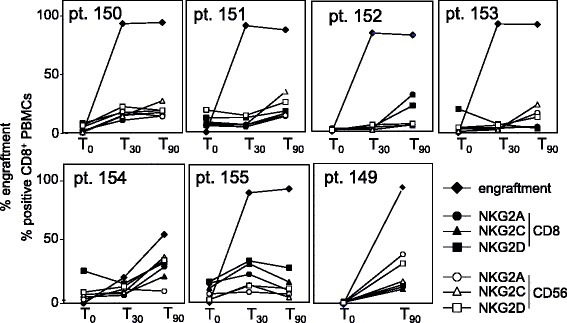
Engraftment and the expression of NKG2A, NKG2C and NKG2D. Percent engraftment (assessed by VNTR) and percent of CD8^+^ and CD56^+^ WBCs positive for NKG2A, NKG2C and NKG2D (double scale in ordinates) were plotted against time elapsed from HSCT

Next, we compared the kinetics of NKG2 receptor up-regulation and engraftment, as assessed by a VNTR assay (see Materials and Methods). Five of the 6 patients tested in a full T_0_-T_30_-T_90_ time course had essentially completed engraftment by day 30 (Fig. 3). NKG2 up-regulation occurred either at the time of engraftment (pts. 150, 155 and the poor-engrafting pt. 154) or later (pts. 151, 152, and 153), clearly showing that it occurs on cells of donor origin. There was no obvious correlation between entity or timing of NKG2 up-regulation and successful engraftment.

### Up-regulation of NKG2A, NKG2C and NKG2D in CD8^+^ T lymphocytes and CD56^+^ NK cells from autotransplanted patients

**Fig. 4:**
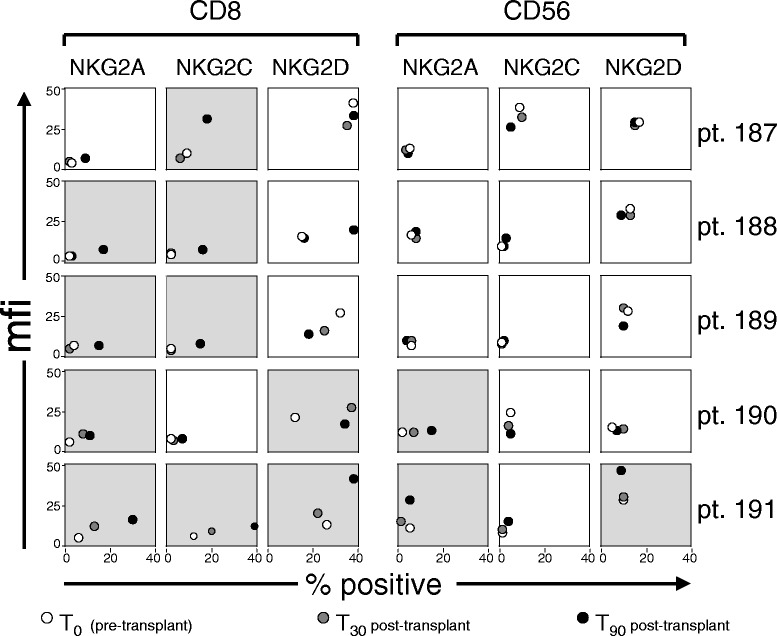
Quantitative evaluation of NKG2A, NKG2C and NKG2D levels following autologous HSCT. Flow cytometry data obtained from 5 patients were graphically elaborated exactly as in Fig. 2. Panels in which NKG2 up-regulation is clearly detectable are in grey

Similar to allotransplantation, autotransplantation resulted in NKG2A, NKG2C and NKG2D up-regulation (increases in both the percentage of positive cells and mfi). However, up-regulation was clearly detected in only 14/30 flow cytometry determinations (Fig. 4, grey panels), most often in CD8^+^ T lymphocytes, and it was invariably of much lesser entity. As a result of weaker and T cell-selective up-regulation, correlation coefficients among NKG2A, NKG2C and NKG2D, calculated as in allotranspanted patients, were weaker than in allotransplantation (Additional file 1: Table S2B). Likewise, CD56 stain increased to a lesser extent and in a minority of autotransplanted patients (Additional file 1: Table S3). These results demonstrate that NKG2 receptor up-regulation, although optimal in an allogeneic setting, also occurs as a result of autotransplantation.

### NKG2 receptor up-regulation and clinical data

The NKG2 activation/inhibition balance was empirically estimated, as described in Additional file 1: Results, by calculating an activation/inhibition ratio, termed R(act/inh). Higher R(act/inh) values identify patients in which the NKG2C/D receptors are up-regulated to an extent greater than NKG2A. When R(act/inh) was plotted vs survival (Additional file 1: Figure S1), values were comprised within a narrow range in 10/12 patients, showing that the NKG2 activation/inhibition balance is similar in spite of a considerable heterogeneity in underlying malignancies, conditioning regimens, engraftment kinetics, infection/clinical course, and allo/auto transplantation settings. This suggests that NKG2 modulation is a standard response to HSCT procedures. Although this study was not designed to address possible influences of NKG2 up-regulation on survival, the only two outliers with high R(act/inh) ratios coincided with the longest and shortest survivors within the allotransplantation and autotransplantation groups (Additional file 1: Figure S1). Pt 155 displayed an immature (CD56-high) post-transplant immunophenotype similar to the other allotransplanted patients. Pt 187 displayed a marginal increase in CD56 mfi similar to autotransplanted pts 189 and 190 (Additional file 1: Table S3). Altogether, high R(act/inh) ratios appear to be the only distinctive feature of pts 155 and 187, in the context of the limited testing performed herein.

### Expression of NKG2 ligands in hematologic malignancies from patients undergoing autotransplantation

**Fig. 5:**
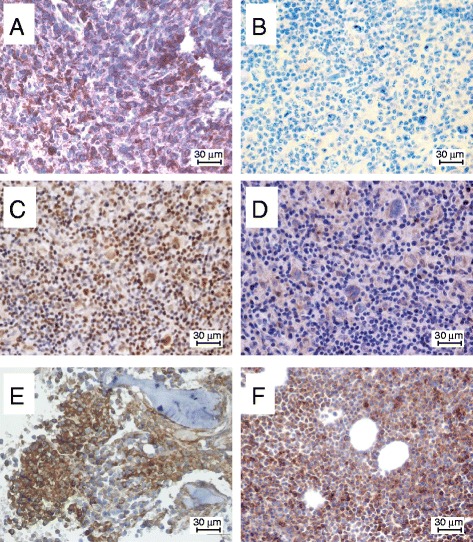
Immunohistochemical staining with mAb MEM-E/02 to HLA-E. HLA-E is strongly expressed in the bone lytic localizations of a Diffuse Large B Cell Lymphoma (panel **a**; pt 188; score = *2*), but not in a case of Hodgkin’s Lymphoma (**b**; pt 190; score = *0*), although isolated cells with histiocytic appearance show weak cytoplasmic stain. The same case of Hodgkin Lymphoma moderately expresses ULBP-1 and ULBP-2 in Hodgkin’s cells and Reed-Sternberg cells (**c** and **d**; score = *1*). Background lymphocytes moderately express ULBP-1 (**c**). A bone marrow biopsy of a Multiple Myeloma is strongly reactive for HLA-E (**e**; pt 191; score = *2*), and this reactivity is enhanced and becomes highly diffuse (**f**; score = *3*) at relapse, 880 days post-transplant. All panels FFPE tissues; 400x magnification

Diagnostic biopsies (available in three cases) were assessed by immunohistochemistry for the expression of HLA-E (the ligand of inhibitory NKG2A and activating NKG2C receptors), and for MICA, ULBP-1, ULBP-2 and ULBP-3 (the ligands of the activating NKG2D receptor), as described in Methods. Representative results are shown in Fig. 5, and a synopsis is provided in Table 3. From 2 to 3 of the 5 tested NKG2 ligands were constitutively expressed, although with different intensities, by tumor cells (and occasionally by background lymphocytes) prior to HSCT (T_0_). In addition, serial bone marrow biopsies were available at four time points for a multiple myeloma: two were obtained before transplantation (T_0_), and two after relapse. One representative lesion of each kind is shown in Fig. 5e and f, and the results are summarized in Table 3. As compared to T_0_ biopsies, both biopsies taken at relapse displayed increased HLA-E stain, and *de novo* appearance of MICA and ULBP-1 expression. Altogether, 4 out of 5 tested NKG2 ligands became expressed in this multiple myeloma after HSCT. These results show that in some patients undergoing autologous HSCT there is a paired up-regulation/high expression of the NKG2A/C/D receptors on circulating immune effectors, and of their cognate tumor immune ligands on hematopoietic malignancies. This suggests potential receptor:ligand interplay *in vivo*.

**Table 3:** Summary of Immunohistochemical testing of neoplastic lesions from autotransplanted patients

	Time	HLA-E	MICA	ULBP-1	ULBP-2	ULBP-3
pt 188 (DLBCL)	T_0_	2	0	1	2	0
pt 190 (HL)	T_0_	0	0	2	2	0
pt 191 (MM)	T_0_	2	0	0	2	0
	Relapse	3	1	1	2	0

*DLBCL* Diffuse Large B cell Lymphoma, *HL* Hodgkin Lymphoma, *MM* Multiple Myeloma

## Discussion

The ability of mature NK cells to be re-educated and adapt to the environment [25–27] provides a solid rationale to their use in HSCT. Interaction of inhibitory NKG2A with self-HLA ligands is a prominent NK cell licencing checkpoint [28]. Accordingly, NKG2A overexpression accompanied by KIR down-regulation, and occasionally CD56 up-regulation, has been concordantly detected in all the available HSCT studies [12–18].

### Simultaneous up-regulation of NKG2A, NKG2C and NKG2D

In the present report, we show that not only inhibitory NKG2A, but also receptors mediating activation (NKG2C and NKG2D) are simultaneously up-regulated in HSCT. It has been shown that activating NKG2 receptors, although expressed at low levels, may overcome the inhibitory NKG2A block in NK cells, contributing to antimicrobial defense [29]. Thus, apart from its potential ability to trigger NK cell maturation and foster GvT, NKG2C/D up-regulation during engraftment may be beneficial to control infection in immunosuppressed patients [30]. As noted by several authors, NK cell receptor plasticity lasts several months, but NKG2A levels return to baseline donor levels thereafter [13, 14]. Accordingly, we have shown herein that engraftment occurs between days 30 and 90 in essentially all the allotransplanted patients, and is accompanied or immediately followed by receptor up-regulation. Based on these data, attempts to turn the balance of dual recognition toward triggering should be made within this time window.

### NKG2 up-regulation on both T and NK cells

It is not surprising that NKG2 up-regulation is generalized, e.g. it involves both CD56^+^ populations (largely including NK cells), as shown by several groups, and CD8^+^ T cells, as shown herein. NK cells integrate opposing influences by default [31], but this functional mode may also be adopted by anti-tumor T lymphocytes [32]. In addition, the classical separation between innate and adaptive immunity has recently been blurred [33]. Differences remain, in that NK cell education/licensing is a dynamic process requiring continuous sampling of MHC class I ligands, whereas positive selection of T cells is a one-time event [25–27, 33]. However, the present observation that NKG2 up-regulation also occurs on engrafting T cells suggests that these may undergo permanent, NK cell-like education/re-tuning. Evidence has indeed been provided that CD94:NKG2C expressed on certain T cell subsets may drive T cell expansion and triggering of effector functions [20]. This is particularly relevant to the autotransplantation setting, in which up-regulation was more marked on T than NK cells. We have recently reviewed the available evidence indicating that it takes both T and NK cells to mount effective antitumor immune responses [34].

### NKG2 up-regulation, engraftment and stress

NKG2 up-regulation either coincides with (beginning on day 30) or follows (day 90) engraftment, as assessed by a VNTR assay. This is consistent with up-regulation taking place on cells of donor origin, as expected. Although access to primary data may be required to draw rigorous conclusions, comparison among the only available report on haploidentical transplantation [14], the numerous studies on HLA-matched allotransplantation [12, 13, 15–18] including the present one, and the present results on autotransplanted patients (not tested before) suggests that NKG2A up-regulation is greatest, intermediate and minimal, respectively, in these three settings. Likewise, CD56 up-regulation, previously detected in allotransplanted patients [14, 16–18], was shown herein to also occur in autotransplanted patients, but to a lesser extent. Thus, there is a gradient: the greater the genetic/phenotypic mismatch, the greater the extent of phenotypic up-regulation. Altogether, the timing and gradient of NKG2 up-regulation suggest the co-existence of immune re-education and a general stress response to pre-transplant chemotherapy and post-transplant re-population. Possibly, chemotherapy without transplantation may give rise to similar effects, as long as immune effectors are given the chance to recover and become re-educated. It will be of interest to assess NKG2 up-regulation in cancer patients with an un-manipulated hematopoietic compartment.

### Implications of NKG2 up-regulation

Although we are not aware of large randomized studies, high persistent expression of inhibitory NKG2A on donor NK cells has been associated with a poor transplantation outcome in HLA-matched [12, 13] and haploidentical [14] HSCT. In the present study, we found that the extent of up-regulation of the individual NKG2 receptors and the ratio of activation vs inhibition were similar in most patients. Therefore, much larger numbers of patients are necessary to determine whether taking into account more receptors might provide additional prognostic information.

However, 2/12 tested patients displayed a clear prevalence of activating receptor expression. It is apparently counterintuitive that these two outliers are also the longest and shortest survivors in the allotransplant (pt. 155) and autotransplant (pt. 187) groups, respectively, and that the former displays a CD56-high post-transplant phenotype suggesting persistence of immature NK cells. Possibly, a prevalence of activating NKG2 receptor expression may have a biological role in the allotransplant setting, in which it occurs on both T and NK cells, but not in the autotransplant setting, in which NKG2 up-regulation is much weaker and T cell-selective. Alternatively, or in addition, receptor engagement with certain tumor ligands may primarily determine the prognosis, since NKG2 ligands were detected in essentially all the tested malignancies, and in one patient they were found to be enhanced on post-transplant relapse. Systematic testing of NKG2 receptors and their tumor ligands is necessary to determine the effect of HSCT on the tumor-host interplay in hematologic malignancies.

Whatever the interpretation, further studies are needed focusing on subsets of HSCT patients with selected hematological diseases. It will be of interest to assess the functional and clinical outcomes of NKG2:ligand interactions after up-regulation.

## Conclusions

In this report, we have shown that activating NKG2C and NKG2D receptors are up-regulated in transplanted immune cells of donor origin as a result of HSCT procedures. Of interest, previous studies have concordantly shown that the *ex vivo* expansion of NK cells turns the NK receptor balance toward activation, although the entity of NKG2C and NKG2D up-regulation differs in different reports [35, 36]. Possibly, this depends at least in part on the source (PBMCs vs cord blood CD34+ stem cells) and protocols (feeder layers of irradiated hematopoietic cells vs cytokines alone) for NK cell expansion. Thus, strategies of *in vitro* expansion and *in vivo* triggering (e.g. by HSCT, as shown herein) of NK cells show both overlapping and complementation, and should be carefully thought out and combined for optimal therapeutic efficacy.

## Additional file

Additional file 1: Supplemental Methods and Results. A supplementary Table (Table S1) summarizing the pertinent literature. A supplemental figure (Fig. S1) displaying correlations between NKG2 up-regulation and survival. References. (PDF 324 kb)

## Abbreviations

HSCT: Hematopoietic stem cell transplantation
NK: Natural killer
KIR: Killer immunoglobulin-like receptors
HLA: Human leukocyte antigens
GvT: Graft versus tumor
GvH: Graft versus host
MICA/B: Major Histocompatibility Class I-like molecules A and B
ULBPs: UL16 Binding Proteins
MAC: Myeloablative conditioning
RIC: Reduced intensity conditioning
WBC: White blood cells
VNTR: Variable nucleotide tandem repeat
QRT-PCR: Quantitative Real-Time PCR
GAPDH: Glyceraldehyde phosphate dehydrogenase
pt.: Patient
FFPE: Formalin fixed paraffin embedded
mfi: Mean fluorescence intensity
R(act/inh): Ratio (activation/inhibition)
AML: Acute myeloid leukemia
B-ALL: B cell acute lymphoid leukemia
DLBCL: Diffuse large B cell lymphoma
MM: Multiple myeloma
HL: Hodgkin’s lymphoma
SAA: Severe aplastic anemia
PB: Peripheral blood
BM: Bone marrow
aGvHD: Acute graft versus host disease
