# Supplementary material for: Up-regulation of activating and inhibitory NKG2 receptors in allogeneic and autologous hematopoietic stem cell grafts
Source: J Exp Clin Cancer Res. 2015 Sep 11;34(1):98. doi: 10.1186/s13046-015-0213-y (PMC4567793; doi:10.1186/s13046-015-0213-y)
Supplement: Additional file 1: — Supplemental Methods and Results. A supplementary Table (Table S1) summarizing the pertinent literature. A supplemental figure (Fig. S1) displaying correlations between NKG2 up-regulation and survival. References. (PDF 324 kb) [file 13046_2015_213_MOESM1_ESM.pdf]

Up-regulation of activating and inhibitory NKG2 receptors in allogeneic and autologous hematopoietic stem cell grafts

Alessandra Picardi, Andrea Mengarelli, Mirella Marino, Enzo Gallo, Maria Benevolo, Edoardo Pescarmona, Roberta Cocco, Rocco Fraioli, Elisa Tremante, Maria Concetta Petti, Paolo De Fabritiis, and Patrizio Giacomini

Additional file 1 – Supplemental Information

Supplemental Methods

Conditioning regimens prior to allotransplantation

Five recipients of allogeneic HSCT (Table I) received myeloablative conditioning regimens (MAC): four of them were treated with oral Busulfan (16 mg/kg) and Cyclophosphamide (120 mg/kg). A case of Severe Aplastic Anemia was treated with Cyclophosphamide (200 mg/kg), and a case of multiple myeloma was treated with fractioned Total Body Irradiation (12 Gy) + Melphalan (140 mg/m^2). The remaining patient received a reduced intensity conditioning (RIC) regimen based on a combination of Thiotepa (10 mg/kg), Fludarabine (60 mg/m^2) and Cyclophosphamide (60 mg/kg), because she had developed multiple comorbidities during previous treatments (Table I). As GvHD prophylaxis, all the patients received a Cyclosporine and Metotrexate short course, intensified with anti-thymocyte globuline (ATG@Fresenius) during the conditioning regimen in the 2 cases transplanted with unrelated grafts.

Variable Nucleotide Tandem Repeat (VNTR) assay

Chimerism was assessed by Quantitative Real-Time PCR (QRT-PCR) amplification of 11 biallelic Variable Nucleotide Tandem Repeats (VNTR) located on chromosomes 1, 6, 9, 11, 17, 18, 20, X

and Y, as described [1], using a LightCycler 2.0 (Roche). The glyceraldehyde phosphate dehydrogenase (GAPDH) gene was included as a non-polymorphic gene. Reference amplification curves were obtained by running in parallel QRT-PCR using as templates mixtures containing known relative amounts of donor and recipient DNAs. Negative controls (no template DNA) were invariably included, and all determinations were performed in triplicate. The intensities of all the donor-specific and recipient-specific amplimers were averaged, and % chimerism was expressed as the relative amount of donor DNA in sample DNA.

Supplemental Results

Simultaneous up-regulation of NKG2A, NKG2C and NKG2D

To determine whether or not NKG2 receptors are simultaneously up-regulated, flow cytometry data were elaborated as follows. First, we multiplied the percent positive by the mfi values of NKG2A, NKG2C and NKG2D flow cytometry determinations. The resulting products were calculated separately for CD8 and CD56 cells at T_0 (before transplant), T_1 (30 days after transplant) and T_2 (90 days after transplant). By this means, a product slant value was assigned to each of the white, grey and black dots visible in Figs. 2 and 4. Next, the Pearson's correlation coefficients were calculated (by Microsoft Excel) among series of three product slant values (NKG2A vs NKG2C; NKG2A vs NKG2D and NKG2C vs NKG2D). The results are reported in Table S2A. From this Table, it may be appreciated that in most cases correlations are strong (>0.7), but weak or even inverse correlations may occasionally be seen. It may be concluded that up-regulation of NKG2A, NKG2C and NKG2D is in most cases simultaneous.

Table S2B reports the same analysis for autotransplanted patients. The results are similar although correlations are weaker.

Increase in CD56 expression post transplant

Table S3 shows the mfi values, Mean values and Standard Deviations of CD56 stain at the T_0 (before transplant) and T_{90} (90 days post-transplant) time points for all the patients. It clearly appears that CD56 stain is brighter at T_{90} than T_0, although a two-tailed Student *t* test (paired) shows that differences in CD56 expression is significant only in allotransplanted patients. CD56 up-regulation, in combination with the phenotypically detectable increases in NKG2 and decrease in KIR2DL expression (see text in the paper), is consistent with an 'immature' NK cell phenotype during engraftment, and with a deeper NK cell retuning in allotransplanted as compared to autotransplanted patients.

NKG2 up-regulation and clinical data

To empirically estimate the activation/inhibition balance, the sum of the slant product values (calculated as described above) of activating receptor expression (NKG2C + NKG2D) was divided by the product values of inhibitory receptor expression (NKG2A). This resulted in 12 ratios, denominated R(activation/inhibition), one for each patient. R(act/inh) values were plotted vs days of Overall Survival (OS), as shown in Fig. S1. A remarkable sample homogeneity was observed across different diseases, conditioning regimens, engraftment kinetics, and even allo/auto transplantation settings, since 6/7 dots in panel A, and 4/5 dots in panel B had similar R(act/inh) values and clustered together. In agreement with a simultaneous and proportional up-regulation of all NKG2 receptors in all the cell subsets, the R(act/inh) scattering of the dots remained remarkably similar even when they were separately calculated for the CD8 and CD56 populations (not shown). Only pt. 155 (multiple myeloma) and pt. 187 (AML) clustered apart due to both high R(act/inh) values, and their longest and shortest survival times within the allotransplantation and autotransplantation groups, respectively (Fig. S1A and S1B). Thus, prevalence of activating receptor expression was observed in these two clinical outliers.

Table S1
Summary of NKG2 studies in HSCT

					Receptor expression in:								
	Disease (case number)	HLA/KIR match[1]			T cells	NK cells							NKG2↑: impact on survival
						KIR	NKG2			NCR (NKp-)			
		haplo	matched	auto			A	C	D	30	44	46	
Shilling et al, 2002 [2]	CML (n=12) AML (n=6)[2]		MR + MU	yes[3]	NT[4]	↓[5]	↑						patterns[6]
Shilling et al, 2003 [3]	CML (n=12) AML (n=6)		MR + MU		NT	↓	↑						patterns[6]
Nguyen et al, 2005 [4]	AML (n=10)	HI			NT	↓	↑		=	d	↑	↑	possibly poor
Vitale et al, 2006 [5]	Miscell (n=25)		MR + MU		NT	↓	↑						
Dulphy et al, 2008 [6]	Miscell (n=43)		MR + MU		NT	↓	↑	=[7]	↑	=	=	↑	
Boyiadzis et al, 2008 [7]	Miscell (n=14)		MR		NT		↑		↑	↑		↑	
Tanaka et al, 2009 [8]	Miscell (n=11)		MU[8]		NT		↓	↑					

[1] HLA Haploidentical (HI); Matched Related (MR); Matched Unrelated (MU); variable (1 to 3 HLA-A, -B, -C loci) mismatch (VMM).
[2] Acute Myelogenous Leukemia (AML), Chronic Myelogenous Leukemia (CML); Non-Hodkin's Lymphoma (NHL)
[3] Non Hodgkin Lymphomas (NHL)
[4] NT : not tested
[5] ↓; ↑; = : down-regulation, up-regulation; no change
[6] Idiosyncratic expression patterns associated with poor outcome
[7] up-regulation trend detected
[8] cord blood transplantation. 4/6 and 5/6 HLA loci matches

Table S2

A: Pearson correlation coefficients for NKG2 up-regulation (allotransplanted patients)

		CD8			CD56		
	NKG2-	A/C	A/D	C/D	A/C	A/D	C/D
Allotransplanted patients	pt 149	NA[1]	NA	NA	NA	NA	NA
	pt 150	0.91	0.93	0.99	0.32	0.93	0.64
	pt 151	0.99	0.99	0.99	0.99	0.99	0.99
	pt 152	0.99	0.98	0.98	0.79	0.97	0.76
	pt 153	0.99	-070	-078	0.99	0.99	0.98
	pt 154	0.99	0.99	0.98	0.65	0.68	0.99
	pt 155	0.84	0.59	0.93	0.89	0.99	0.84

[1] NA: not assessed because the series comprises only two values (T_0 and T_{90})

B: Pearson correlation coefficients for NKG2 up-regulation (autotransplanted patients)

		CD8			CD56		
	NKG2-	A/C	A/D	C/D	A/C	A/D	C/D
Autotransplanted patients	187	0.99	0.04	0.08	0.53	0.92	0.82
	188	0.99	0.99	0.99	0.99	-0.99	-0.99
	189	0.98	-0.55	-0.68	0.99	-0.95	-0.95
	190	0.75	0.69	0.04	-0.68	0.07	-0.77
	191	0.99	0.98	0.98	0.94	0.91	0.99

Table S3

CD56 mean fluorescence intensities of PBMCs from allotransplanted and autotransplanted patients at T_0 (before transplant) and T_{90} (day 90 after transplant).

		CD56 mfi	
		T_0	T_{90}
**allotransplanted patients**	pt 149	28	55
	pt 150	40	60
	pt 151	44	90
	pt 152	25	35
	pt 153	29	26
	pt 154	37	46
	**pt 155**	18	45
**MEAN (SD)**		31.6 (9.1)	51.0 (20.6)
***t* test**		**0.018**	
**autotransplanted patients**	**pt 187**	45	50
	pt 188	47	93
	pt 189	50	54
	pt 190	45	50
	pt 191	60	80
**MEAN (SD)**		49.4 (6.3)	65.4 (19.9)
***t* test**		0.118	

Figure S1

NKG2 up-regulation and clinical data

R(act/inh) vs Overall Survival (OS) for allotransplanted and autotransplanted patients are separately displayed in panels A and B, respectively. For R(act/inh) calculation see Supplemental Results. Each dot represents a single patient, identified by number. Two outliers are indicated.

References

1. Jimenez-Velasco A, Barrios M, Roman-Gomez J, Navarro G, Buno I, Castillejo JA et al. Reliable quantification of hematopoietic chimerism after allogeneic transplantation for acute leukemia using amplification by real-time PCR of null alleles and insertion/deletion polymorphisms. Leukemia. 2005;19:336-43.

2. Shilling HG, Young N, Guethlein LA, Cheng NW, Gardiner CM, Tyan D et al. Genetic control of human NK cell repertoire. J Immunol. 2002;169:239-47.

3. Shilling H, McQueen K, Cheng N, Shizuru J, Negrin R, Parham P. Reconstitution of NK cell receptor repertoire following HLA-matched hematopoietic cell transplantation. Blood. 2003;101:3730-40.

4. Nguyen S, Dhedin N, Vernant J, Kuentz M, Al Jijakli A, Rouas-Freiss N et al. NK-cell reconstitution after haploidentical hematopoietic stem-cell transplantations: immaturity of NK cells and inhibitory effect of NKG2A override GvL effect. Blood. 2005;105:4135-42.

5. Vitale C, Pitto A, Benvenuto F, Ponte M, Bellomo R, Frassoni F et al. Phenotypic and functional analysis of the HLA-class I-specific inhibitory receptors of natural killer cells isolated from peripheral blood of patients undergoing bone marrow transplantation from matched unrelated donors. Hematol J. 2006;1:136-44.

6. Dulphy N, Haas P, Busson M, Belhadj S, Peffault de Latour R, Robin M et al. An unusual CD56(bright) CD16(low) NK cell subset dominates the early posttransplant period following HLA-matched hematopoietic stem cell transplantation. J Immunol. 2008;181:2227-37.

7. Boyiadzis M, Memon S, Carson J, Allen K, Szczepanski MJ, Vance BA et al. Up-regulation of NK cell activating receptors following allogeneic hematopoietic stem cell transplantation under a lymphodepleting reduced intensity regimen is associated with elevated IL-15 levels. Biol Blood Marrow Transplant. 2008;14:290-300.

8. Tanaka J, Sugita J, Asanuma S, Arita K, Shono Y, Kikutchi M et al. Increased number of CD16(+)CD56(dim) NK cells in peripheral blood mononuclear cells after allogeneic cord blood transplantation. Human Immunol. 2009;70(9):701-5. doi:10.1016/j.humimm.2009.06.002.

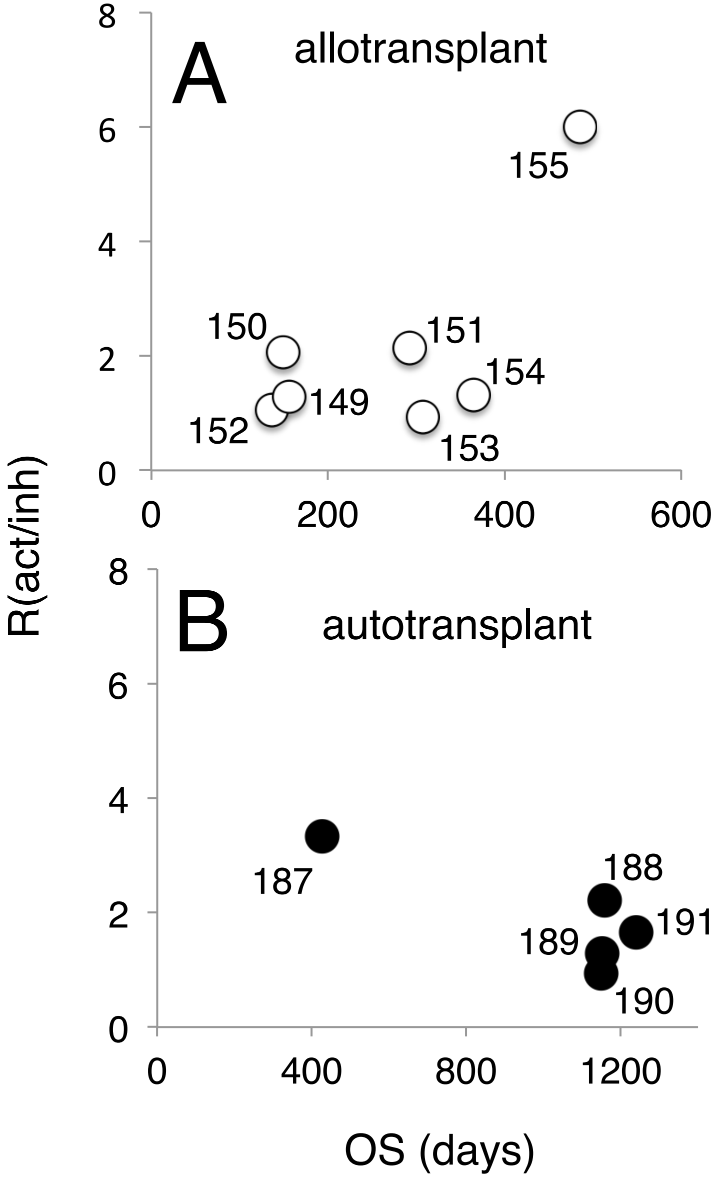

A
allotransplant
8
6
4
2
0
155
150
151
154
149
152
153
0
200
400
600
R(act/inh)
B
autotransplant
8
6
4
2
0
187
188
191
189
190
0
400
800
1200
OS (days)

Fig. S1
